# The psychological impact of COVID-19 on health care workers working in a unique environment under the umbrella of Qatar Red Crescent Society

**DOI:** 10.1016/j.heliyon.2021.e07236

**Published:** 2021-06-05

**Authors:** Muna Abed Alah, Khaled Ali, Sami Abdeen, Ghadir Al-Jayyousi, Hasan Kasem, Feroz Poolakundan, Shafik Al-Mahbshii, Iheb Bougmiza

**Affiliations:** a Community Medicine Department, Hamad Medical Corporation (HMC), Doha, Qatar; b Department of Public Health, College of Health Sciences, QU Health, Qatar University, Doha, Qatar; c Medical Affairs Division, Qatar Red Crescent Society (QRCS), Qatar; d Community Medicine Department, Primary Health Care Corporation (PHCC), Doha, Qatar; e Community Medicine Department, College of Medicine, Sousse University, Tunisia

**Keywords:** Anxiety, COVID-19, Depression, Health care worker, Qatar Red Crescent, Stress

## Abstract

**Objectives:**

to determine the levels of depression, anxiety, and stress among healthcare workers (HCWs) working in a unique male-dominated environment under the umbrella of Qatar Red Crescent, and to explore the associated factors during COVID-19 pandemic in Qatar.

**Methods:**

a cross-sectional study utilizing a web-based survey was conducted in the period between 15 November 2020 and 22 December 2020. Depression, anxiety, and stress were determined using the 9-items patient health questionnaire (PHQ-9), the 7-item generalized anxiety disorder (GAD-7) scale, and the 22- item impact of event scale revised (IES-R), respectively. We conducted multivariable logistic regression analysis to determine the predictors of mental health outcomes among HCWs.

**Results:**

the proportions of the participants reporting symptoms of depression, anxiety, and stress were 12.4 (95%CI: 9.3–16.1), 14.2 (95%CI: 10.9–18.1), and 18.5% (95%CI: 14.8–22.7) respectively. Arabs had significantly more severe anxiety levels than non-Arabs (p = 0.031), HCWs with high COVID-19 risk perception experienced more severe mental health outcomes (p < 0.001). The multivariable logistic regression showed that high risk perception was independently associated with depression (adjusted OR 4.62, 95%CI: 2.00–10.68), anxiety (adjusted OR 4.90, 95%CI: 2.24–10.68), and stress (adjusted OR 3.067, 95%CI: 1.62–5.79) with p < 0.001. Compared to nurses, technicians and paramedics were more likely to report anxiety symptoms with (adjusted OR 2.97, 95%CI: 1.23–7.17, p = 0.015), and (adjusted OR 5.48, 95%CI: 1.86–16.12, p = 0.002) respectively. Having a relative or a friend died of COVID-19 infection was significantly associated with depression symptoms (adjusted OR 2.54, 95%CI: 1.21–5.36, p = 0.014). Not living with family was significantly associated with the presence of different mental health outcomes.

**Conclusion:**

relatively lower rates of mental health outcomes in this study compared to others could have several explanations related to the unique characteristics of our target population and their working environment. Ensuring proper mental health support for HCWs is highly recommended.

## 1 Introduction

During infectious outbreaks, when health systems are overwhelmed, frontline health care workers (HCWs) experience an increasing workload and they are at increased risk of infection; therefore, they are more susceptible to complex emotional reactions and psychological distress. Evidence from previous infectious outbreaks such as Severe Acute Respiratory Syndrome (SARS) (Chong et al., 2004; Tam et al., 2004), Middle East Respiratory Syndrome (MERS-CoV) (Khalid et al., 2016), and H1N1 influenza pandemic (Goulia et al., 2010), indicated that HCWs are at risk of physical and psychological harm. Similarly, emerging evidence is suggesting that the Corona Virus Disease (COVID-19) pandemic as a stressful event can significantly affect mental and psychological wellbeing of HCWs (Al Maqbali et al., 2021; Mahmud et al., 2021).

Mental health problems of HCWs would impair their attention and adversely affect their cognitive functioning and clinical decision making (Leblanc, 2009). Several studies have assessed the psychological impact of COVID-19 pandemic on HCWs. A systematic review and metanalysis of 27 studies assessing the effects of the COVID-19 pandemic on the mental health of HCWs particularly in relation to the prevalence of depression, anxiety, and stress showed a pooled prevalence of 39.8, 41.3, and 54.3% respectively (Mahmud et al., 2021). In China, studies conducted in this area showed that a considerable proportion of HCWs reported symptoms of depression, anxiety, and distress (Lai et al., 2020; Liang et al., 2020; Que et al., 2020). In USA, 13.9, 15.6, and 22.8% of HCWs in one study had a probable major depression (MD), generalized anxiety disorder (GAD), and post-traumatic stress disorder (PTSD) respectively (Hennein et al., 2021). Variable levels of anxiety and depression were also detected among HCWs in Poland, Italy, Kenya, and Turkey (Araç and Dönmezdil, 2020; Gorini et al., 2020; Onchonga et al., 2021; Wańkowicz et al., 2020). A high level of physical symptoms reflecting somatization was also detected in a study from Singapore (Ng et al., 2020b). In Egypt over 70% of HCWs experienced depression, anxiety and stress symptoms (Elkholy et al., 2020). In Qatar, two studies that assessed mental wellbeing as measured by the Warwick-Edinburgh Mental Well-Being Scale (WEMWBS) of HCWs showed that 17.4%, and 30% of the participants had well-being scores of less than 45, indicating suboptimal wellbeing and a high risk of psychological distress and depression (Wadoo et al, 2021a, 2021b).

Several factors determine how HCWs respond to different psychological stressors such as the occupational role of the HCW (Du et al., 2020; Guo et al., 2020; Koh et al., 2005; Lai et al., 2020; Maunder et al., 2004; Nickell et al., 2004; Wadoo et al., 2021b; Wong et al., 2005; Zhu et al., 2020), specialized training and previous experience working during crises (Maunder et al., 2006; Wong et al., 2007), being a high risk (frontline) worker (i.e. directly working with suspected or confirmed cases) (Abolfotouh et al., 2017; Chen et al., 2005; Guo et al., 2020; Lai et al., 2020; Maunder et al., 2004; McAlonan et al., 2007; Poon et al., 2004; Tam et al., 2004; Wu et al., 2009; Zhu et al., 2020), adequacy and perceived effectiveness of personal protective equipment (PPE) and infection and prevention control measures (Khalid et al., 2016; Marjanovic et al., 2007; Zhu et al., 2020), perceived risk of infecting friends, colleagues or family members (Khalid et al., 2016; Tam et al., 2004; Zhu et al., 2020), adequacy of organizational support during outbreaks (Marjanovic et al., 2007; Tam et al., 2004; Zhu et al., 2020), social rejection or isolation (people avoiding HCWs or their families in relation to their work during outbreaks) (Koh et al., 2005; Nickell et al., 2004; Zhu et al., 2020), and socio-demographic characteristics of HCWs such as age, gender, nationality, and parental status (Abolfotouh et al., 2017; Lai et al., 2020; Nickell et al., 2004; Zhu et al., 2020).

Established in March 1978 as Qatar's first volunteering charitable organization, Qatar Red Crescent Society (QRCS) is an active member of the International Red Cross and Red Crescent Movement. It works to empower communities to protect their safety and dignity in the face of humanitarian crises and disasters such as natural disasters, conflicts, or widespread epidemics. It provides a wide range of relief and humanitarian activities including food aids, urgent accommodation, water and sanitation services, and emergency healthcare in the form of mobile clinics, medical and emergency teams, emergency health centers and camps, and it serves a wide range of medical workers including physicians, nurses, first responders, technicians, and disaster preparedness and relief workers both in and out of Qatar with diverse training courses in compliance with the highest quality standards including lifeguard, disaster management, first aid and CPR courses (QRCS: Qatar Red Crescent Society, 2017). During the current pandemic, QRCS is supporting Qatar and other countries in the fight against COVID-19 by collecting funds to support COVID-19 control efforts. They made remarkable progress in supporting 22 countries to reduce the spread of the pandemic by providing personal protective equipment (PPE) for volunteers and community to reduce the virus dissemination, launching community awareness campaigns about COVID-19, and training health professionals at isolation centers and providing them with hygiene and nonmedical kits (gulf times, 2020). Medical services provided by QRCS include emergency medical services where QRCS operates a fleet of 50 well-equipped ambulance vehicles. Additionally, the medical affairs division of QRCS is responsible for the provision of health care services to single male worker population including craft and manual workers (CMW) through designated primary health care facilities being referred to as workers health centers. A total of four operational health centers are currently providing these services to over 700.000 visitors per year and are run by only males HCWs (QRCS: Qatar Red Crescent Society, 2017). The medical workforce of QRCS consists of about 550 HCWs. The COVID-19 epidemic in Qatar disproportionately affected the CMW population who comprises 60% of the total population (Al Kuwari et al., 2020). Thus, we can speculate that HCWs running workers health centers are at a particularly higher risk of contracting the infection dealing with this vulnerable population, compared to other HCWs, and hence are more susceptible to psychological distress.

Studies assessing the psychological impact of COVID-19 on HCWs in the Arabian Gulf region are limited. The aim of our study is to explore the impact of COVID-19 pandemic on the mental health of HCWs working under the umbrella of QRCS running workers health centers and associated factors. To our knowledge, there have been no studies evaluating mental health outcomes of a similar population of HCWs. We believe that the results of this study can guide the development of effective health care interventions and strategies to mitigate the impact of infectious outbreaks and other public health emergencies on the mental wellbeing of HCWs. Our objectives are to determine the levels of depression, anxiety, and stress among HCWs working in a unique male-dominated environment under the umbrella of QRCS using validated measurement tools and to explore the associated factors during COVID-19 pandemic in Qatar.

## 2 Methods

### 2.1 Study design, and the target population

A cross-sectional study utilizing a web-based survey was conducted among HCWs working under the umbrella of QRCS. Data were collected in the period between 15 November 2020 and 22 December 2020. Our target population included all full time HCWs regardless of their positions, working in workers’ health centers of QRCS. Volunteers were not included. This study was approved by the Qatar University Institutional Review Board (Approval No. QU-IRB 1398-E/20). Other necessary approvals were obtained from QRCS.

### 2.2 Data collection

Because of the low response rate generally encountered in web-based surveys and in order to improve the external validity of our study, we invited all eligible HCWs (550) to take the survey. They were contacted via e-mail with an information letter and a link to the electronic version of the questionnaire that was developed using Google forms software. The letter stated the purpose of the study and that the participation is voluntary. Taking the survey implied informed consent. Participants were free to terminate the survey at any time they desired. The survey was anonymous, and confidentiality of information was assured. Weekly reminders were sent to maximize the response rate. To guard against duplicate responses, we asked the supervisors in QRCS to instruct HCWs to take the survey only once.

### 2.3 Overview of the survey

We developed the survey in English utilizing a validated and reliable psychological assessment tools. The face and content validity were assured by experts in the field. The survey was piloted on a sample of 15 HCWs who were excluded from the sample. Refinements were made according to feedback from the pilot study. The survey consisted of five sections. The first one assessed the socio-demographic characteristics for the participants (age, gender, nationality, clinical experience, area of work… etc), history of chronic diseases, in addition to some COVID-19 related information such as the frequency of dealing with suspected or confirmed cases, and the status of training on the use of personal protective equipment (PPE) or emergency preparedness for infectious outbreaks. The second section addressed COVID-19 risk perception. Third, fourth and fifth sections assessed mental health outcomes (depression, anxiety and stress) via three valid and reliable psychological scales: the 9-item patient health questionnaire (PHQ-9), the 7-item generalized anxiety disorder (GAD-7) scale, and the 22- item impact of event scale revised (IES-R), respectively. PHQ-9 and GAD-7 assess how often the participant experienced symptoms of depression or anxiety respectively in the past 2 weeks, each item is scored on a scale from 0 to 3: 0 (not at all sure), 1 (several days), 2 (over half the days), 3 (nearly every day). IES-R is used as a self-report measure of current subjective distress in response to a specific traumatic event. It is composed of 22 items that represent difficulties people sometimes have after stressful life events. Participants were asked to report the degree of distress experienced for each item in the past 7 days. The 5 points on the scale are: 0 (not at all), 1 (a little bit), 2 (moderately), 3 (quite a bit), 4 (extremely).

### 2.4 Study variables

Our main focus was on detecting symptoms of depression, anxiety and stress using validated and reliable tools. The total scores of these measurement tools were interpreted based on values established in the literature as follows: PHQ-9, normal to minimal depression (0–4), mild (5–9), moderate (10–14), moderately severe to severe (15–27) (Kroenke et al., 2001; Lai et al., 2020); a cut-off 10 was found to have the best diagnostic properties for detecting major depressive disorder (Levis et al., 2019; Manea et al., 2012). GAD-7, normal to minimal anxiety (0–4), mild (5–9), moderate (10–14), and severe (15–21) (Lo, 2008; Spitzer et al., 2006) a cut-off score of 10 has sensitivity of 89% and a specificity of 82% in diagnosing of GAD (Spitzer et al., 2006). For IES-R a cut-off 24 will be of clinical concern, and a cut-off of 33 represent the best diagnostic accuracy for post-traumatic stress disorder (PTSD) (Creamer et al., 2003; “The Impact of Event Scale – Revised (IES-R) – NovoPsych Psychometrics,” n.d.).

To assess COVID-19 risk perception, participants were asked to indicate their degrees of agreement on a four-point scale with nine statements. The four points on the scale are 0 (strongly disagree), 1 (disagree), 2 (agree), 3 (strongly agree). Examples of statements include: “I feel that I am at a greater risk of getting infected with COVID-19 because of my work”, “I think of resigning because of the COVID-19 outbreak”, “I feel that my family are at a greater risk of COVID-19 infection because of my work”, “Protective measures such as PPE are not effective in protecting me from COVID”. A total score was calculated by summing the scores of the nine statements, then taking the median score (10) as a cut-off to classify the results into high-risk perception (score ≥10), and low risk perception (score <10).

### 2.5 Statistical analysis

Data analysis was performed using the Statistical Package for the Social Sciences (SPSS) version 26. Descriptive statistics were presented as frequencies and percentages for categorical variables. After testing for normality and taking into account the severity levels of mental health outcomes as ordinal dependent variables, the nonparametric Mann-Whitney U test and Kruskal-Wallis test were applied to compare the severity levels of each symptom between two or more groups. To determine potential predictors for the presence of symptoms of depression, anxiety, and stress in the participants, multivariable logistic regression analysis was performed, and the associations between risk factors and outcomes are presented as adjusted odds ratios (ORs) and 95%CIs. Goodness of fit was assessed using Hosmer and Lemeshow test. P-values less than 0.05 are considered significant.

### 2.6 Ethical considerations

This study was approved by the Qatar University Institutional Review Board (Approval No. QU-IRB 1398-E/20). Other necessary approvals were obtained from QRCS. A letter attached with the survey stated the purpose of the study, assured confidentiality of information collected, participants’ anonymity, and their right to discontinue at any point of the study with no consequences. The acceptance to participate implied informed consent of the participant. This study complies with all necessary regulations.

## 3 Results

### 3.1 Sociodemographic characteristics

A link to the online survey was sent to 550 healthcare workers, 394 completed the survey, giving a response rate of 71.6%. All were men aged 21–62 years (Median age 34, IQR 32–40). Of the participants, the majority were nurses (45.9%), non-Arab (84.3%), with 70% of them being Indians. Majority (87.6%) were married, 73.4% lived with family member/s while only 12.7% lived alone. 22.8% of the participants worked in emergency settings.14.7% had one chronic disease (most commonly hypertension or Diabetes Mellitus), and only five (1.3%) had two or more chronic diseases (Table 1).

**Table 1:** Sociodemographic profiles and background information of the participants.

Characteristic		No. (%)
**Age categories**	20–29	16 (4.1)
	30–39	274 (69.5)
	40–49	89 (22.6)
	≥50	15 (3.8)
**Nationality**	Arab∗	62 (15.7)
	Non-Arab†	332 (84.3)
**Marital Status**	Married	345 (87.6)
	Unmarried	49 (12.4)
**Parental Status**	No children	95 (24.1)
	One child	122 (31.0)
	Two or more children	177 (44.9)
**Living with**	Family member/s	289 (73.4)
	Friends	55 (14.0)
	Alone	50 (12.7)
**Profession**	Physician	101 (25.6)
	Nurse	181 (45.9)
	Technician	75 (19.0)
	Paramedic	37 (9.4)
**Area of work**	Emergency	90 (22.8)
	Non-emergency	304 (77.2)
**Clinical experience**	Less than 5 years	16 (4.1)
	5 years or more	378 (95.9)
**Presence of chronic diseases**	None	331 (84.0)
	1 chronic disease‡	58 (14.7)
	2 or more chronic diseases‡	5 (1.3)

∗ Includes Syrian, Jordanian, Egyptian, Sudanese, Bahraini, Tunisian, Yemeni, and Palestinian.

† Includes Indian, Filipino, and American.

‡ Chronic diseases include Hypertension, Diabetes, Asthma, Cardiovascular Disease, Hypothyroidism, Dyslipidemia, Atrial Fibrillation and Inflammatory bowel diseases.

Of the participants, 280 (71.1%) reported dealing with suspected or confirmed COVID-19 cases frequently. Also, 365 (92.6%) have received training on the use of PPE, and 183 (46.4%) have received emergency preparedness training for infectious diseases outbreaks.

### 3.2 Severity of mental health outcomes and associated factors

Proportions of participants reporting symptoms of depression (PHQ-9 score ≥5), anxiety (GAD-7 score ≥5), and stress (IES-R score ≥24) were 12.4 (95%CI: 9.3–16.1), 14.2 (95%CI: 10.9–18.1), and 18.5% (95%CI: 14.8–22.7) respectively. Participants who met the diagnostic cut-off for depression (PHQ-9 score ≥10) were 17 (4.3%), for anxiety (GAD-7 score ≥10) were also 17 (4.3%, 95%CI: 2.5–6.8) and for PTSD (IES-R score ≥33) were 38 (9.6%, 95%CI: 6.9–13). Only 10 (2.54%, 95%CI: 1.2–4.6) participants met the diagnostic cut-offs for the three scales together. Arabs had significantly more severe anxiety levels than non-Arabs (p = 0.031). Paramedics had more severe anxiety levels compared to nurses (p = 0.005), while technicians showed a significantly more severe stress levels than nurses (p = 0.014). Compared with participants with low-risk perception of COVID-19 infection, those with high-risk perception (perception score ≥10) experienced more severe depression (p < 0.001), anxiety (p < 0.001) and stress (p = 0.001) levels. Those who had a relative or a friend who died as a result of COVID-19 infection were more likely to report more severe symptoms of depression (p < 0.001) and stress (p = 0.035) compared to those who did not (Table 2).

**Table 2:** Severity levels of Depression, Anxiety and Stress among different subgroups.

Severity Categories		Totals No. (%)	Nationality No. (%)		*P* value	Profession No. (%)				*P* value	Area of work No. (%)		*P* value	PPE training No. (%)		*P* value	Emergency preparedness training No. (%)		*P* value	Having relatives or friend died of COVID-19 No. (%)		*P* value	Risk perception of COVID-19 infection No. (%)		*P* value
			Arab (n = 62)	Non-Arab (n = 332)		Physician (n = 101)	Nurse (n = 181)	Technician (n = 75)	Paramedic (n = 37)		Emergency (n = 90)	Non-Emergency (n = 304)		Yes (n = 365)	No (n = 29)		Yes (n = 183)	No (n = 211)		Yes (n = 181)	No (n = 213)		Low (n = 186)	High (n = 208)	
**Depression (PHQ-9)**	Normal/minimal	345 (87.6)	52 (83.9)	293 (88.3)	0.273	86 (85.1)	162 (89.5)	67 (89.3)	30 (81.1)	0.311	76 (84.4)	269 (88.5)	0.275	318 (87.1)	27 (93.1)	0.353	155 (84.7)	190 (90.0)	0.087	147 (81.2)	198 (93.0)	<0.001	177 (95.2)	168 (80.8)	<0.001
	Mild	32 (8.1)	4 (6.5)	28 (8.4)		8 (7.9)	17 (9.4)	5 (6.7)	2 (5.4)		8 (8.9)	24 (7.9)		31 (8.5)	1 (3.4)		16 (8.7)	16 (7.6)		21 (11.6)	11 (5.2)		9 (4.8)	23 (11.1)	
	Moderate	7 (1.8)	2 (3.2)	5 (1.5)		3 (3)	1 (0.6)	2 (2.7)	1 (2.7)		1 (1.1)	6 (2.0)		6 (1.6)	1 (3.4)		3 (1.6)	4 (1.9)		5 (2.8)	2 (0.9)		0 (0.0)	7 (3.4)	
	Moderately severe -severe	10 (2.5)	4 (6.5)	6 (1.8)		4 (4)	1 (0.6)	1 (1.3)	4 (10.8)		5 (5.6)	5 (1.6)		10 (2.7)	0 (0.0)		9 (4.9)	1 (0.5)		8 (4.4)	2 (0.9)		0 (0.0)	10 (4.8)	
**Anxiety (GAD-7)**	Normal/minimal	338 (85.8)	48 (77.4)	290 (87.3)	0.031	84 (83.2)	166 (91.7)	61 (81.3)	27 (73)	0.005∗	76 (84.4)	262 (86.2)	0.633	313 (85.8)	25 (86.2)	0.999	151 (82.5)	187 (88.6)	0.071	149 (82.3)	189 (88.7)	0.055	175 (94.1)	163 (78.4)	<0.001
	Mild	39 (9.9)	8 (12.9)	31 (9.3)		10 (9.9)	13 (7.2)	11 (14.7)	5 (13.5)		8 (8.9)	31 (10.2)		37 (10.1)	2 (6.9)		20 (10.9)	19 (9.0)		20 (11.0)	19 (8.9)		9 (4.8)	30 (14.4)	
	Moderate	10 (2.5)	3 (4.8)	7 (2.1)		3 (3)	1 (0.6)	2 (2.7)	4 (10.8)		5 (5.6)	5 (1.6)		9 (2.5)	1 (3.4)		8 (4.4)	2 (0.9)		6 (3.3)	4 (1.9)		2 (1.1)	8 (3.8)	
	Severe	7 (1.8)	3 (4.8)	4 (1.2)		4 (4)	1 (0.6)	1 (1.3)	1 (2.7)		1 (1.1)	6 (2.0)		6 (1.6)	1 (3.4)		4 (2.2)	3 (1.4)		6 (3.3)	1 (0.5)		0 (0.0)	7 (3.4)	
**Stress (IES-R)**	Score >24	321 (81.5)	49 (79)	272 (81.9)	0.496	84 (83.2)	157 (86.7)	53 (70.7)	27 (73)	0.014∗	70 (77.8)	251 (82.6)	0.243	295 (80.8)	26 (89.7)	0.299	139 (76.0)	182 (86.3)	0.009	140 (77.3)	181 (85.0)	0.035	164 (88.2)	157 (75.5)	0.001
	Score 24-32	35 (8.9)	4 (6.5)	31 (9.3)		5 (5)	13 (7.2)	13 (17.3)	4 (10.8)		7 (7.8)	28 (9.2)		35 (9.6)	0 (0.0)		21 (11.5)	14 (6.6)		16 (8.8)	19 (8.9)		15 (8.1)	20 (9.6)	
	Score ≥33	38 (9.6)	9 (14.5)	29 (8.7)		12 (11.9)	11 (6.1)	9 (12)	6 (16.2)		13 (14.4)	25 (8.2)		35 (9.6)	3 (10.3)		23 (12.6)	15 (7.1)		25 (13.8)	13 (6.1)		7 (3.8)	31 (14.9)	

∗ After doing pairwise comparisons between the profession groups using the Dunn-Bonferroni approach (post-hoc test for Kruskal Wallis test) it was found that there is statistically significant difference in anxiety severity levels between nurses and paramedics groups and in Stress severity levels between nurses and technicians groups.

### 3.3 Predictors of mental health outcomes

A logistic regression was carried out to assess the effects of independent variables such as age, nationality, area of work, profession, COVID-19 risk perception and others on the likelihood of having depression symptoms (PHQ-9 score ≥5), anxiety symptoms (GAD-7 score ≥5), and stress symptoms (IES-R score ≥24). Selection of independent variables to be included in the model was based on clinical and statistical relevance. All models were statistically significant (p < 0.001) when compared to the null model with chi-square values (χ2) of 62.765, 63.470, 60.085 for depression, anxiety and stress models respectively, explained 27.9, 26.6, and 22.9% (Nagelkerke R^2^) of the variation of depression, anxiety, and stress symptoms and correctly predicted 89.3, 81.5, and 83.5% of cases, respectively. Hosmer and Lemeshow test was done to assess the goodness of fit of our models and indicated that our models fits the data better than the null models. COVID-19 risk perception was found to be an independent risk factor for all psychiatric symptoms (p < 0.001). Participants with high-risk perception were more likely to develop symptoms of depression, anxiety and stress. Profession was significantly associated with the presence of anxiety (p = 0.009) and stress symptoms (p = 0.005). Compared to nurses, technicians and paramedics were more likely to report anxiety symptoms with p-values of (0.015,0.002) and adjusted ORs of (2.97, 95%CI: 1.23–7.17), (5.48, 95%CI: 1.86–16.12) respectively and more likely to report stress symptoms with p-values of (0.001, 0.037) and adjusted OR (3.60, 95%CI: 1.72–7.56), (2.90, 95%CI: 1.06–4.90) respectively. Compared to those living with their families, participants living alone were more likely to report depression symptoms (p = 0.018, adjusted OR 3.57, 95%CI: 1.25–10.20), and those living with their friends were more likely to develop stress symptoms (p = 0.008, adjusted OR 4.06, 95%CI: 1.43–11.51). Having a relative or a friend died of COVID-19 infection was significantly associated with depression symptoms (p = 0.014, adjusted OR 2.54, 95% CI: 1.21–5.36) but not with anxiety (p = 0.287) or stress symptoms (p = 0.122). Unexpectedly, those who received training on emergency preparedness for infectious outbreaks were more likely to have stress symptoms compared to those who did not (p = 0.011). No significant associations were found between (age, nationality, marital and parental status, area of work, clinical experience, frequency of dealing with suspected or confirmed COVID-19 cases, presence of chronic diseases and previous training on PPE) and the presence of the depression, anxiety, and stress symptoms (Table 3).

**Table 3:** Predictors of mental health outcomes as identified by multivariable logistic regression analysis.

Variable		Depressive symptoms (PHQ-9 ≥ 5)			Anxiety symptoms (GAD-7 ≥ 5)			Stress symptoms (IES-R ≥ 24)		
		No. of symptomatic cases (%)	AOR∗ (95% CI)	*p*-value	No. of symptomatic cases (%)	AOR∗ (95% CI)	*p*-value	No. of symptomatic cases (%)	AOR∗ (95% CI)	*p*-value
**Living with**	Family member/s (n = 289)	28 (9.7)	1 [reference]		36 (12.5)	1 [reference]		43 (14.9)	1 [reference]	
	Friends (n = 55)	9 (16.4)	2.33 (0.62–8.77)	0.221	10 (18.2)	1.66 (0.44–6.20)	0.454	19 (34.5)	4.06 (1.43–11.51)	0.008
	Alone (n = 50)	12 (24.0)	3.57 (1.25–10.20)	0.018	10 (20.0)	1.43 (0.49–4.21)	0.517	11 (22.0)	1.57 (0.62–3.93)	0.341
**Profession**	Nurse (n = 181)	19 (10.5)	1 [reference]		15 (8.3)	1 [reference]		24 (13.3)	1 [reference]	
	Physician (n = 101)	15 (14.9)	2.03 (0.68–6.05)	0.202	17 (16.8)	2.18 (0.74–6.73)	0.156	17 (16.8)	1.70 (0.6–4.47)	0.283
	Technician (n = 75)	8 (10.7)	1.12 (0.42–2.98)	0.818	14 (18.7)	2.97 (1.23–7.17)	0.015	22 (29.3)	3.60 (1.72–7.56)	0.001
	Paramedic (n = 37)	7 (18.9)	2.35 (0.718–7.70)	0.158	10 (27.0)	5.48 (1.86–16.12)	0.002	10 (27.0)	2.90 (1.06–7.90)	0.037
**Area of work**	Non-emergency (n = 304)	35 (11.5)	1 [reference]		42 (13.8)	1 [reference]		53 (17.4)	1 [reference]	
	Emergency (n = 90)	14 (15.6)	1.03 (0.45–2.34)	0.940	14 (15.6)	0.729 (0.33–1.62)	0.438	20 (22.2)	0.943 (0.473–1.88)	0.868
**Risk Perception of COVID-19 infection**	Low perception (n = 186)	9 (4.8)	1 [reference]		11 (5.9)	1 [reference]		22 (11.8)	1 [reference]	
	High perception (n = 208)	40 (19.2)	4.62 (2.00–10.68)	<0.001	45 (21.6)	4.90 (2.24–10.68)	<0.001	51 (24.5)	3.067 (1.62–5.79)	0.001
**Having relatives/friends died with COIVD19**	No (n = 213)	15 (7.0)	1 [reference]		24 (11.3)	1 [reference]		32 (15.0)	1 [reference]	
	Yes (n = 181)	34 (18.8)	2.54 (1.20–5.36)	0.014	32 (17.7)	1.46 (0.73–6.37)	0.287	41 (22.7)	1.61 (0.88–2.96)	0.122
**Emergency preparedness training**	No (n = 211)	21 (10.0)	1 [reference]		24 (11.4)	1 [reference]		29 (13.7)	1 [reference]	
	Yes (n = 183)	28 (15.3)	1.74 (0.85–3.60)	0.135	32 (17.5)	1.8 (0.90–3.61)	0.099	44 (24.0)	2.23 (1.20–4.16)	0.011

Abbreviations: AOR, adjusted odds ratio; CI, confidence interval.

∗ Adjusted for variables in Table 1 as appropriate.

## 4 Discussion

To best of our knowledge this is one of the few studies to address the impact of COVID-19 on mental health of HCWs during COVID-19 in the Middle East and particularly in the Arabian Gulf region, which will add value to the preexisting literature that emerged mostly from China. Studying mental health of HCWs and the factors influencing it would provide evidence to plan for effective interventions to enhance mental health wellbeing for HCWs and lessen the burden on the health care system.

This cross-sectional survey enrolled 394 HCWs out of 550, giving a response rate of about 72%, reflecting the effectiveness of frequent reminders sent to encourage participants to the take the survey. All published studies in this area so far have involved both males and females with varying proportions, unlike our study that included only male HCWs and this can be justified by the fact that QRCS hires only male HCWs to serve in workers health centers as such centers restrict their services to single male workers population including craft and manual workers (CMW). CMW are economically disadvantaged. They live in overcrowded conditions where physical distancing is not feasible rendering them more vulnerable to contract and spread COVID-19 infection. Evidence have shown that this pandemic disproportionately affected racial/ethnic minorities as well as the poor who live in urban settings with more crowded living conditions and higher chances of being employed in public-facing occupations (eg, services and transportation) that would prevent physical distancing (Webb Hooper et al., 2020).

Prevalence rates of depression, anxiety and stress symptoms were found to be 12.4, 14.2, and 18.5% respectively in our study which are much lower than those reported by other studies that used the same measurement tools and the same cut-off points for reporting results, ranging from 53.6%-89% (Elkholy et al., 2020; Liang et al., 2020; Onchonga et al., 2021; Que et al., 2020; Wańkowicz et al., 2020) for depression, 35–92% for anxiety (Elkholy et al., 2020; Liang et al., 2020; Onchonga et al., 2021; Prasad et al., 2020; Que et al., 2020; Wańkowicz et al., 2020), and 71.5% for stress (Lai et al., 2020). This can be explained by the fact that those studies with higher prevalence rates were conducted between February and May 2020 when much more uncertainties were surrounding the pandemic as compared to the time when we conducted our study by the end of 2020, back then people including HCWs became more used to the situation and the number of COVID-19 cases has dropped significantly in Qatar. The epidemiologic curve of COVID-19 cases in Qatar shows how the number of new cases started to decline slowly and gradually in June 2020, reaching very low levels in November and December 2020 (coinciding with the time of our survey), then started to rise again early in January 2021 (Ritchie et al., 2020). However, this explanation might not apply in other populations where levels of mental health outcomes remained high in a post movement lockdown assessment among university HCWs in Malaysia after the government lifted the movement control order (Woon et al., 2020). Other explanations for the relatively lower rates in our study could be, first, in our study only male HCWs were involved as QRCS finds it more convenient to hire only men to run workers health centers, which are designated for single male workers patients. Working in a male-dominated environment for a long period of time might affect the way they perceive and deal with stressors and the way they control and express their emotions. Second, HCWs in this study are working under the umbrella of an international charitable organization that works on helping vulnerable communities, this might indicate that the inherent characteristics of HCWs who serve in such organizations are somehow different than others and might render them more resistant to stressors. In compliance with The International Federation of Red Cross and Red Crescent Societies (IFRC) Strategy 2020, red crescent organizations worldwide aim to effectively contribute to building resilience, which is the ability of individuals, communities, organizations, or countries exposed to disasters, crises, and underlying vulnerabilities to anticipate, reduce the impact of, cope with, and recover from the effects of adversity without compromising their long-term prospects (The International Federation of Red Cross and Red Crescent Societies (IFRC), 2012). Improving resilience among individuals including HCWs might explain the lower rates of mental health outcomes among HCWs in QRCS. A recently published article showed a significant correlation between the level of resilience and anxiety experienced by HCWs during the COVID-19 pandemic. The lower the resilience, the higher the anxiety experienced (Setiawati et al., 2021). QRCS offers a wide range of high-quality trainings for HCWs in and out of Qatar including their staff in the field of first aid and disaster management which we believe will further contribute to building their resilience and helping them adapt with various stressors including those imposed by the pandemic. Additionally, Ministry of Public health (MOPH) in Qatar has established a clear evidence-based infection prevention and control guidelines including the appropriate use of personal protective equipment and different precautions to be followed by different segments of the population including HCWs (Ministry of Public Health Qatar, 2020a). With such clear instructions and guidelines in place, we believe that HCWs would feel safer and more confident when dealing with COVID-19 suspected or confirmed cases or high-risk populations lessening the psychological distress encountered in such situations. MOPH has also established a free and confidential mental health and wellbeing helpline (16000) that is staffed by a team of mental health professionals who can support callers from different categories including HCWs with regard to mental health related issues during COVID-19 pandemic (Ministry of Public Health Qatar, 2020b). Lastly, our study showed that over 80% of participating HCWs are living with their families. Getting social support from their family members might also explain the lower rates of adverse mental health outcomes among them. It is evident from the literature that spirituality and religion are protective factors for physical and mental health (Zimmer et al., 2016). During the current pandemic, spirituality helped many people to make sense of what was happening and not to feel lost in the face of the radical change in the way of living and conducting social relationships (Coppola et al., 2021). We believe that in a multinational country like Qatar hosting people with different religious backgrounds, investigating the role of religion and spirituality in protecting against adverse mental health outcomes during challenging times such as the current pandemic is needed and should be highlighted in depth in future research. Similar rates of depression and anxiety ranging from 11-15% for depression, and 13–16% for anxiety were reported in studies from China and United States (Chew et al., 2020; Evanoff et al., 2020). However, we cannot conclude that those results are comparable to ours as they were based on a different measurement tool, which is Depression, Anxiety and Stress scale -21 items (DASS 21).

Our study showed that having a relative or a friend died of COVID-19 infection was significantly associated with depressive symptoms (p = 0.014), but not with anxiety or stress, while Zhu et al. 2020 found a similar association with anxiety (Zhu et al., 2020). Unsurprisingly, high COVID-19 risk perception was found to be an independent risk factor for all mental health outcomes in our study (p < 0.001). Similar associations were reported in studies from China and Italy (Gorini et al., 2020; Zhu et al., 2020). We found that living alone is a predictor of having depressive symptoms compared to living with family and this is consistent with other studies that reported significant associations between lack of social support and depression (Du et al., 2020; Elbay et al., 2020; Naser et al., 2020). This might indicate that being away from family and unable to travel back home due to travel restriction further contributes to the adverse psychological impact of the pandemic. This might also signify that remote interactions using social media -despite being helpful for many- cannot replace face to face communications for others. This survey showed that technicians and paramedics are more likely to report symptoms of anxiety (p-values of 0.015, 0.002 respectively) and stress (p-values of 0.001, 0.037 respectively) when compared to nurses. One explanation could be that paramedics are more likely to be exposed to emergency cases since their role is to provide advanced emergency medical care for critical and emergent patients, and thus, they are more likely to be exposed to the severe cases of COVID-19 infection. And technicians including lab technicians are directly dealing with infectious samples from suspected or confirmed COVID-19 cases which might put them in a great deal of stress. On the other hand, no significant differences were found between physicians and nurses, inconsistent with other studies where nurses were more likely to report stress than physicians (Lai et al., 2020; Zhu et al., 2020). We did not find any significant associations between (age, nationality, marital and parental status, area of work, clinical experience, frequency of dealing with suspected or confirmed COVID-19 cases, presence of chronic diseases and previous training on PPE) and the presence of the depression, anxiety, or stress symptoms. While other studies did (Elbay et al., 2020; Huang and Zhao, 2020; Lai et al., 2020; Lu et al., 2020; Zhu et al., 2020). Clinical experience of more than 10 years, and concomitant chronic diseases were found significantly associated with stress in one study (Zhu et al., 2020). Another study showed that frontline HCWs were associated with higher risk of symptoms of depression, anxiety, and stress (Lai et al., 2020). Screening for mental health problems in populations of concern such as HCWs should be a priority in healthcare planning for the current and any potential future public health emergencies. Provision of psychosocial care to affected individuals should be integrated in emergency preparedness plans. Maintenance of an optimum mental health is also a responsibility of people themselves. They should be accountable for their actions and emotional wellbeing. Sometimes, simple self help techniques such as biofeedback and mindfulness could be critically beneficial during these challenging times of the pandemic (Sidi, 2020). Additionally, an emphasis must be given for timely psychological support which could take many forms including telemental health services, such as the use of psychiatric teleconsultation to provide support to those in need, and social support groups (Ng et al., 2020a).

### 4.1 Strengths and limitations

This study has several strengths. First, it is one of the few studies addressing the mental health impact of COVID-19 on HCWs in the Arabian Gulf Region and in Qatar. Second, we achieved an unexpectedly high response rate of over 70% despite utilizing a web-based survey. Third, our study is one of the few studies assessing the association between COVID-19 risk perception and mental health outcomes. Also, we targeted a special population of HCWs not studied before, involving those working under the umbrella of QRCS a part of an international charitable organization, consisting of only male HCWs working in a male-dominated environment and providing health care services to a disproportionally high-risk population for COVID-19 infection that involves CMWs. However, involving only male HCWs might compromise the generalizability of the findings, which can be viewed as a limitation for this study. Another limitation might result from the cross-sectional design of this study that does not allow us to follow those with severe psychological symptoms to refer them for help and provide the needed psychological support for them.

### 4.2 Conclusion

COVID-19 is a stressful event impacting the mental health of HCWs. COVID-19 risk perception is an independent risk factor for all mental health outcomes. Relatively lower rates of depression, anxiety and stress in this study compared to others could have several explanations including the stage of the pandemic at the time of collecting data, and the unique characteristics of our target population and their working environment. Nevertheless, these figures can't be ignored. Ensuring proper mental health support for HCWs and implementing tailored interventions are important components of public health measures for addressing COVID-19 pandemic and are highly recommended.

## Declarations

### Author contribution statement

Muna Abed Alah: Conceived and designed the experiments; Performed the experiments; Analyzed and interpreted the data; Contributed reagents, materials, analysis tools or data; Wrote the paper.

Khaled Ali, Sami Abdeen: Performed the experiments; Analyzed and interpreted the data; Contributed reagents, materials, analysis tools or data; Wrote the paper.

Ghadir Al-Jayyousi, Iheb Bougmiza: Analyzed and interpreted the data; Wrote the paper.

Hasan Kasem, Feroz Poolakundan, Shafik Al-Mahbshii: Performed the experiments; Wrote the paper.

### Funding statement

The publication of the article was funded by Qatar National Library.

### Data availability statement

Data will be made available on request.

### Declaration of interest statement

The authors declare no conflict of interest.

### Additional information

No additional information is available for this paper.

## References

[bib1] Abolfotouh M.A., AlQarni A.A., Al-Ghamdi S.M., Salam M., Al-Assiri M.H., Balkhy H.H. (2017). An assessment of the level of concern among hospital-based health-care workers regarding MERS outbreaks in Saudi Arabia. BMC Infect. Dis..

[bib2] Al Kuwari H.M., Abdul Rahim H.F., Abu-Raddad L.J., Abou-Samra A.B., Al Kanaani Z., Al Khal A., Al Kuwari E., Al Marri S., Al Masalmani M., Al Romaihi H.E., Al Thani M.H., Coyle P.V., Latif A.N., Owen R., Bertollini R., Butt A.A. (2020). Epidemiological investigation of the first 5685 cases of SARS-CoV-2 infection in Qatar, 28 February-18 April 2020. BMJ Open.

[bib3] Al Maqbali M., Al Sinani M., Al-Lenjawi B. (2021). Prevalence of stress, depression, anxiety and sleep disturbance among nurses during the COVID-19 pandemic: a systematic review and meta-analysis. J. Psychosom. Res..

[bib4] Araç S., Dönmezdil S. (2020). Investigation of mental health among hospital workers in the COVID-19 pandemic: a cross-sectional study. Sao Paulo Med. J..

[bib5] Chen C.S., Wu H.Y., Yang P., Yen C.F. (2005). Psychological distress of nurses in Taiwan who worked during the outbreak of SARS. Psychiatr. Serv..

[bib6] Chew N.W.S., Lee G.K.H., Tan B.Y.Q., Jing M., Goh Y., Ngiam N.J.H., Yeo L.L.L., Ahmad A., Ahmed Khan F., Napolean Shanmugam G., Sharma A.K., Komalkumar R.N., Meenakshi P.V., Shah K., Patel B., Chan B.P.L., Sunny S., Chandra B., Ong J.J.Y., Paliwal P.R., Wong L.Y.H., Sagayanathan R., Chen J.T., Ying Ng A.Y., Teoh H.L., Tsivgoulis G., Ho C.S., Ho R.C., Sharma V.K. (2020). A multinational, multicentre study on the psychological outcomes and associated physical symptoms amongst healthcare workers during COVID-19 outbreak. Brain Behav. Immun..

[bib7] Chong M.Y., Wang W.C., Hsieh W.C., Lee C.Y., Chiu N.M., Yeh W.C., Huang T.L., Wen J.K., Chen C.L. (2004). Psychological impact of severe acute respiratory syndrome on health workers in a tertiary hospital. Br. J. Psychiatry.

[bib8] Coppola I., Rania N., Parisi R., Lagomarsino F. (2021). Spiritual well-being and mental health during the COVID-19 pandemic in Italy. Front. Psychiatr..

[bib9] Creamer M., Bell R., Failla S. (2003). Psychometric properties of the impact of event scale - revised. Behav. Res. Ther..

[bib10] Du J., Dong L., Wang T., Yuan C., Fu R., Zhang L., Liu B., Zhang M., Yin Y., Qin J., Bouey J., Zhao M., Li X. (2020). Psychological symptoms among frontline healthcare workers during COVID-19 outbreak in Wuhan. Gen. Hosp. Psychiatr..

[bib11] Elbay R.Y., Kurtulmuş A., Arpacıoğlu S., Karadere E. (2020). Depression, anxiety, stress levels of physicians and associated factors in Covid-19 pandemics. Psychiatr. Res..

[bib12] Elkholy H., Tawfik F., Ibrahim I., Salah El-din W., Sabry M., Mohammed S., Hamza M., Alaa M., Fawzy A.Z., Ashmawy R., Sayed M., Omar A.N. (2020). Mental health of frontline healthcare workers exposed to COVID-19 in Egypt: a call for action. Int. J. Soc. Psychiatr..

[bib13] Evanoff B.A., Strickland J.R., Dale A.M., Hayibor L., Page E., Duncan J.G., Kannampallil T., Gray D.L. (2020). Work-related and personal factors associated with mental well-being during the COVID-19 response: survey of health care and other workers. J. Med. Internet Res..

[bib15] Gorini A., Fiabane E., Sommaruga M., Barbieri S., Sottotetti F., La Rovere M.T., Tremoli E., Gabanelli P. (2020). Mental health and risk perception among Italian healthcare workers during the second month of the Covid-19 pandemic. Arch. Psychiatr. Nurs..

[bib16] Goulia P., Mantas C., Dimitroula D., Mantis D., Hyphantis T. (2010). General hospital staff worries, perceived sufficiency of information and associated psychological distress during the A/H1N1 influenza pandemic. BMC Infect. Dis..

[bib17] gulf times (2020). QRCS Backs Covid-19 Control Efforts in Six Countries. https://www.gulf-times.com/story/690971/QRCS-backs-Covid-19-control-efforts-in-six-countri.

[bib18] Guo J., Liao L., Wang B., Li X., Guo L., Tong Z., Guan Q., Zhou M., Wu Y., Zhang J., Gu Y. (2020). Psychological effects of COVID-19 on hospital staff: a national cross-sectional survey of China mainland. SSRN Electron. J..

[bib19] Hennein R., Mew E.J., Lowe S.R. (2021). Socio-ecological predictors of mental health outcomes among healthcare workers during the COVID-19 pandemic in the United States. PloS One.

[bib20] Huang Y., Zhao N. (2020). Generalized anxiety disorder, depressive symptoms and sleep quality during COVID-19 outbreak in China: a web-based cross-sectional survey. Psychiatr. Res..

[bib21] Khalid I., Khalid T.J., Qabajah M.R., Barnard A.G., Qushmaq I.A. (2016). Healthcare workers emotions, perceived stressors and coping strategies during a MERS-CoV outbreak. Clin. Med. Res..

[bib22] Koh D., Lim M.K., Chia S.E., Ko S.M. (2005). Risk perception and impact of severe acute respiratory syndrome ( SARS ) on work and personal lives of. Med. Care.

[bib23] Kroenke K., Spitzer R.L., Williams J.B.W. (2001). The PHQ-9: validity of a brief depression severity measure. J. Gen. Intern. Med..

[bib24] Lai J., Ma S., Wang Y., Cai Z., Hu J., Wei N., Wu J., Du H., Chen T., Li R., Tan H., Kang L., Yao L., Huang M., Wang H., Wang G., Liu Z., Hu S. (2020). Factors associated with mental health outcomes among health care workers exposed to coronavirus disease 2019. JAMA Netw. Open.

[bib25] Leblanc V.R. (2009). The effects of acute stress on performance: implications for health professions education. Acad. Med..

[bib26] Levis B., Benedetti A., Thombs B.D. (2019). Accuracy of Patient Health Questionnaire-9 (PHQ-9) for screening to detect major depression: individual participant data meta-analysis. BMJ.

[bib27] Liang Y., Wu K., Zhou Yongjie, Huang X., Zhou Yueyue, Liu Z. (2020). Mental health in frontline medical workers during the 2019 novel coronavirus disease epidemic in China: a comparison with the general population. Int. J. Environ. Res. Publ. Health.

[bib28] Lo B. (2008). Validation and standardization of the generalized anxiety disorder screener ( GAD-7 ) in the. Gen. Popul..

[bib29] Lu W., Wang H., Lin Y., Li L. (2020). Psychological status of medical workforce during the COVID-19 pandemic: a cross-sectional study. Psychiatr. Res..

[bib30] Mahmud S., Hossain S., Muyeed A., Islam M.M., Mohsin M. (2021). The global prevalence of depression, anxiety, stress, and, insomnia and its changes among health professionals during covid-19 pandemic: a rapid systematic review and meta-analysis. SSRN Electron. J..

[bib31] Manea L., Gilbody S., McMillan D. (2012). Optimal cut-off score for diagnosing depression with the Patient Health Questionnaire (PHQ-9): a meta-analysis. CMAJ (Can. Med. Assoc. J.).

[bib32] Marjanovic Z., Greenglass E.R., Coffey S. (2007). The relevance of psychosocial variables and working conditions in predicting nurses’ coping strategies during the SARS crisis: an online questionnaire survey. Int. J. Nurs. Stud..

[bib33] Maunder R.G., Lancee W.J., Balderson K.E., Bennett J.P., Borgundvaag B., Evans S., Fernandes C.M.B., Goldbloom D.S., Gupta M., Hunter J.J., Hall L.M.G., Nagle L.M., Pain C., Peczeniuk S.S., Raymond G., Read N., Rourke S.B., Steinberg R.J., Stewart T.E., VanDeVelde-Coke S., Veldhorst G.G., Wasylenki D.A. (2006). Long-term psychological and occupational effects of providing hospital healthcare during SARS outbreak. Emerg. Infect. Dis..

[bib34] Maunder R.G., Lancee W.J., Rourke S., Hunter J.J., Goldbloom D., Balderson K., Petryshen P., Steinberg R., Wasylenki D., Koh D., Fones C.S.L. (2004). Factors associated with the psychological impact of severe acute respiratory syndrome on nurses and other hospital workers in Toronto. Psychosom. Med..

[bib35] McAlonan G.M., Lee A.M., Cheung V., Cheung C., Tsang K.W.T., Sham P.C., Chua S.E., Wong J.G.W.S. (2007). Immediate and sustained psychological impact of an emerging infectious disease outbreak on health care workers. Can. J. Psychiatr..

[bib36] Ministry of Public Health Qatar (2020). NATIONAL INFECTION PREVENTION AND CONTROL INTERIM GUIDELINE FOR COVID-19.

[bib37] Ministry of Public Health Qatar (2020). COVID-19 National Mental Health. https://sehanafsia.moph.gov.qa/English/Pages/COVID19_NMH_Helpline.aspx.

[bib38] Naser A.Y., Dahmash E.Z., Al-Rousan R., Alwafi H., Alrawashdeh H.M., Ghoul I., Abidine A., Bokhary M.A., AL-Hadithi H.T., Ali D., Abuthawabeh R., Abdelwahab G.M., Alhartani Y.J., Al Muhaisen H., Dagash A., Alyami H.S. (2020). Mental health status of the general population, healthcare professionals, and university students during 2019 coronavirus disease outbreak in Jordan: a cross-sectional study. Brain Behav..

[bib39] Ng Q.X., Chee K.T., De Deyn M.L.Z.Q., Chua Z. (2020). Staying connected during the COVID-19 pandemic. Int. J. Soc. Psychiatr..

[bib40] Ng Q.X., De Deyn M.L.Z.Q., Lim D.Y., Chan H.W., Yeo W.S. (2020). The wounded healer: a narrative review of the mental health effects of the COVID-19 pandemic on healthcare workers. Asian J. Psychiatr..

[bib41] Nickell L.A., Crighton E.J., Tracy C.S., Al-Enazy H., Bolaji Y., Hanjrah S., Hussain A., Makhlouf S., Upshur R.E.G. (2004). Psychosocial effects of SARS on hospital staff: survey of a large tertiary care institution. CMAJ (Can. Med. Assoc. J.).

[bib42] Onchonga D., Ngetich E., Makunda W., Wainaina P., Wangeshi D., viktoria P. (2021). Anxiety and depression due to 2019 SARS-CoV-2 among frontier healthcare workers in Kenya. Heliyon.

[bib43] Poon E., Liu K.S., Cheong D.L., Lee C.K., Yam L.Y.C., Tang W.N. (2004). Impact of severe acute respiratory syndrome on anxiety levels of frontline health care workers. Hong Kong Med. J..

[bib44] Prasad A., Civantos A.M., Byrnes Y., Chorath K., Poonia S., Chang C., Graboyes E.M., Bur A.M., Thakkar P., Deng J., Seth R., Trosman S., Wong A., Laitman B.M., Shah J., Stubbs V., Long Q., Choby G., Rassekh C.H., Thaler E.R., Rajasekaran K. (2020). Snapshot impact of COVID-19 on mental wellness in nonphysician otolaryngology health care workers: a national study. OTO Open.

[bib45] QRCS: Qatar Red Crescent Society (2017). Annual Reports. https://www.qrcs.org.qa/General/AnnualReport.

[bib46] Que J., Shi L., Deng J., Liu J., Zhang L., Wu S., Gong Y., Huang W., Yuan K., Yan W., Sun Y., Ran M., Bao Y., Lu L. (2020). Psychological impact of the covid-19 pandemic on healthcare workers: a cross-sectional study in China. Gen. Psychiatr..

[bib47] Ritchie H., Ortiz-Ospina E., Beltekian D., Mathieu E., Hasell J., Macdonald B., Giattino C., Appel C., Rodés-Guirao L., Roser M. (2020). Coronavirus Pandemic (COVID-19). https://ourworldindata.org/coronavirus/country/qatar.

[bib48] Setiawati Y., Wahyuhadi J., Joestandari F., Maramis M.M., Atika A. (2021). Anxiety and resilience of healthcare workers during COVID-19 pandemic in Indonesia. J. Multidiscip. Healthc..

[bib49] Sidi H. (2020). The psychological sequelae during mental health and COVID-19 pandemic: learning from the past for today’s coping styles. Med. Health.

[bib50] Spitzer R.L., Kroenke K., Williams J.B.W., Lo B. (2006). A Brief Measure for Assessing Generalized Anxiety Disorder.

[bib51] Tam C.W.C., Pang E.P.F., Lam L.C.W., Chiu H.F.K. (2004). Severe acute respiratory syndrome (SARS) in Hongkong in 2003: stress and psychological impact among frontline healthcare workers. Psychol. Med..

[bib52] The impact of event scale – revised (IES-R) – NovoPsych Psychometrics. https://novopsych.com.au/assessments/the-impact-of-event-scale-revised-ies-r/.

[bib53] The International Federation of Red Cross and Red Crescent Societies (IFRC) (2012). The Road to Resilience Bridging Relief and Development for a More Sustainable Future IFRC Discussion Paper on Resilience-June 2012. http://www.ifrc.org.

[bib54] Wadoo O., Latoo J., Iqbal Y., Chandrappa N.S.K., Chandra P., Masoodi N.A., Al-Maslamani M.A.R.S., Alabdulla M. (2021). Mental wellbeing of frontline healthcare workers during COVID-19 pandemic in Qatar. Asian J. Psychiatr..

[bib55] Wadoo O., Latoo J., Iqbal Y., Kudlur Chandrappa N.S., Chandra P., Masoodi N.A., Rahman S., Al-Maslamani M.A., Alabdulla M. (2021). Mental wellbeing of healthcare workers working in quarantine centers during the COVID-19 pandemic in Qatar. Qatar Med. J..

[bib56] Wańkowicz P., Szylińska A., Rotter I. (2020). Assessment of mental health factors among health professionals depending on their contact with covid-19 patients. Int. J. Environ. Res. Publ. Health.

[bib57] Webb Hooper M., Nápoles A.M., Pérez-Stable E.J. (2020). COVID-19 and racial/ethnic disparities. JAMA, J. Am. Med. Assoc..

[bib58] Wong T.W., Yau J.K.Y., Chan C.L.W., Kwong R.S.Y., Ho S.M.Y., Lau C.C., Lau F.L., Lit C.H. (2005). The psychological impact of severe acute respiratory syndrome outbreak on healthcare workers in emergency departments and how they cope. Eur. J. Emerg. Med..

[bib59] Wong W.C.W., Wong S.Y.S., Lee A., Goggins W.B. (2007). How to provide an effective primary health care in fighting against severe acute respiratory syndrome: the experiences of two cities. Am. J. Infect. Contr..

[bib60] Woon L.S.C., Sidi H., Jaafar N.R.N., Bin Abdullah M.F.I.L. (2020). Mental health status of university healthcare workers during the covid-19 pandemic: a post–movement lockdown assessment. Int. J. Environ. Res. Publ. Health.

[bib61] Wu P., Fang Y., Guan Z., Fan B., Kong J., Yao Z., Liu X., Fuller C.J., Susser E., Lu J., Hoven C.W. (2009). The psychological impact of the SARS epidemic on hospital employees in China: exposure, risk perception, and altruistic acceptance of risk. Can. J. Psychiatr..

[bib62] Zhu Z., Ph D., Xu S., Ph D., Wang H., Med M., Liu Z., Ph D. (2020). COVID-19 in Wuhan : Immediate Psychological Impact on 5062 Health Workers.

[bib63] Zimmer Z., Jagger C., Chiu C.T., Ofstedal M.B., Rojo F., Saito Y. (2016). Spirituality, religiosity, aging and health in global perspective: a review. SSM - Popul. Heal..

